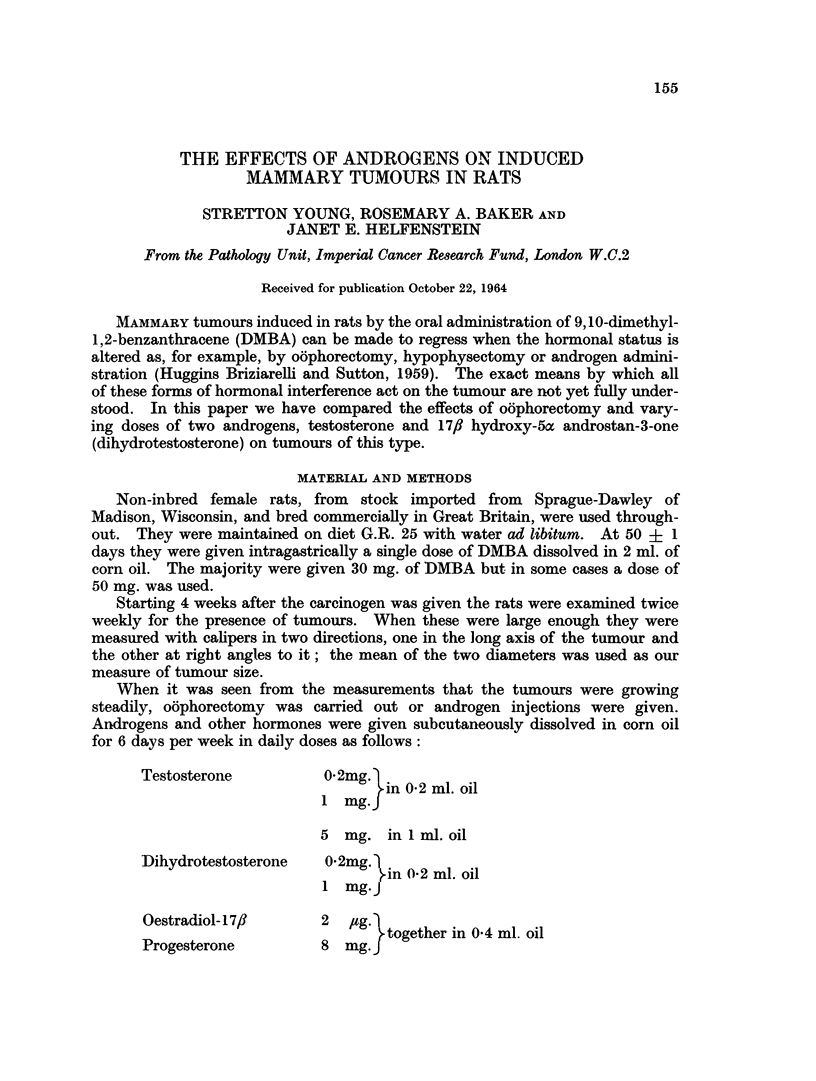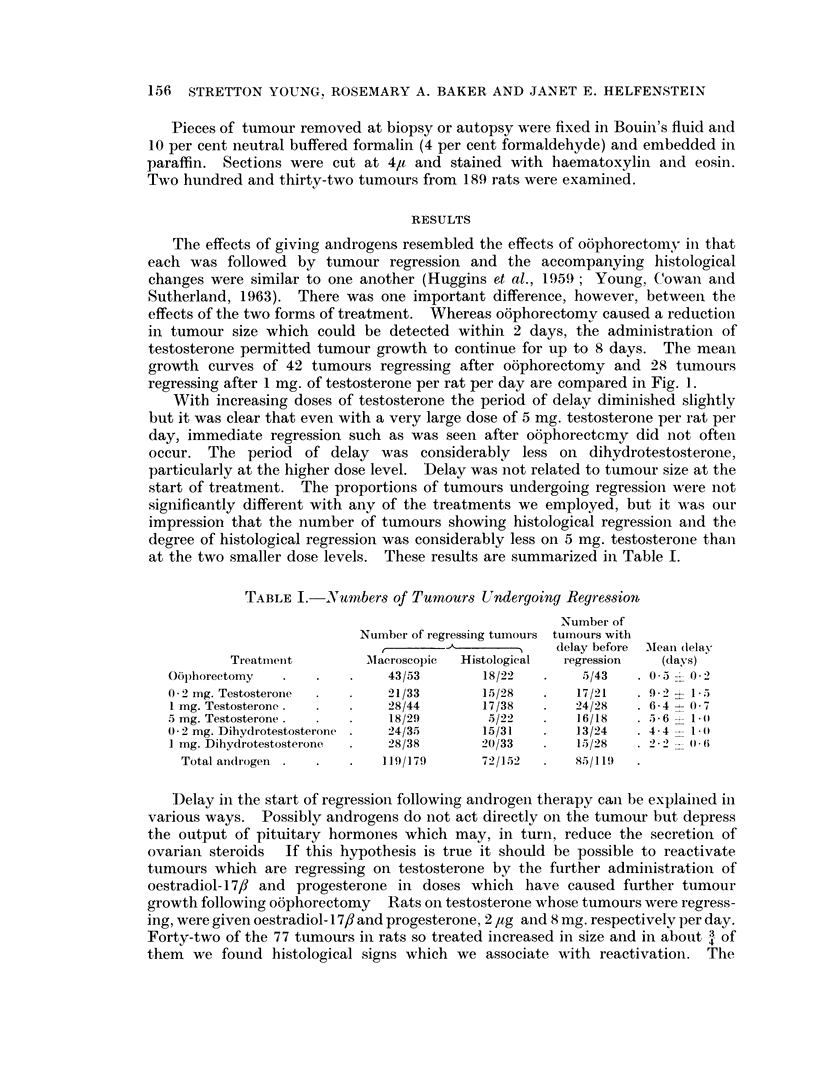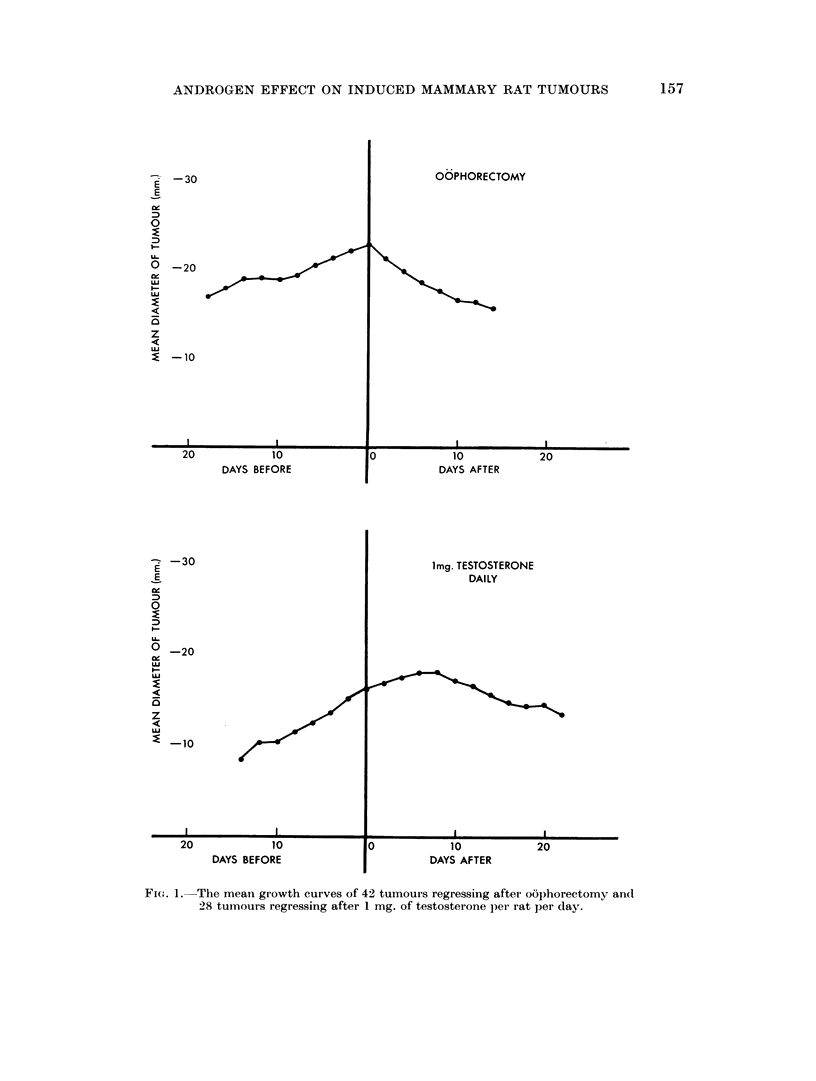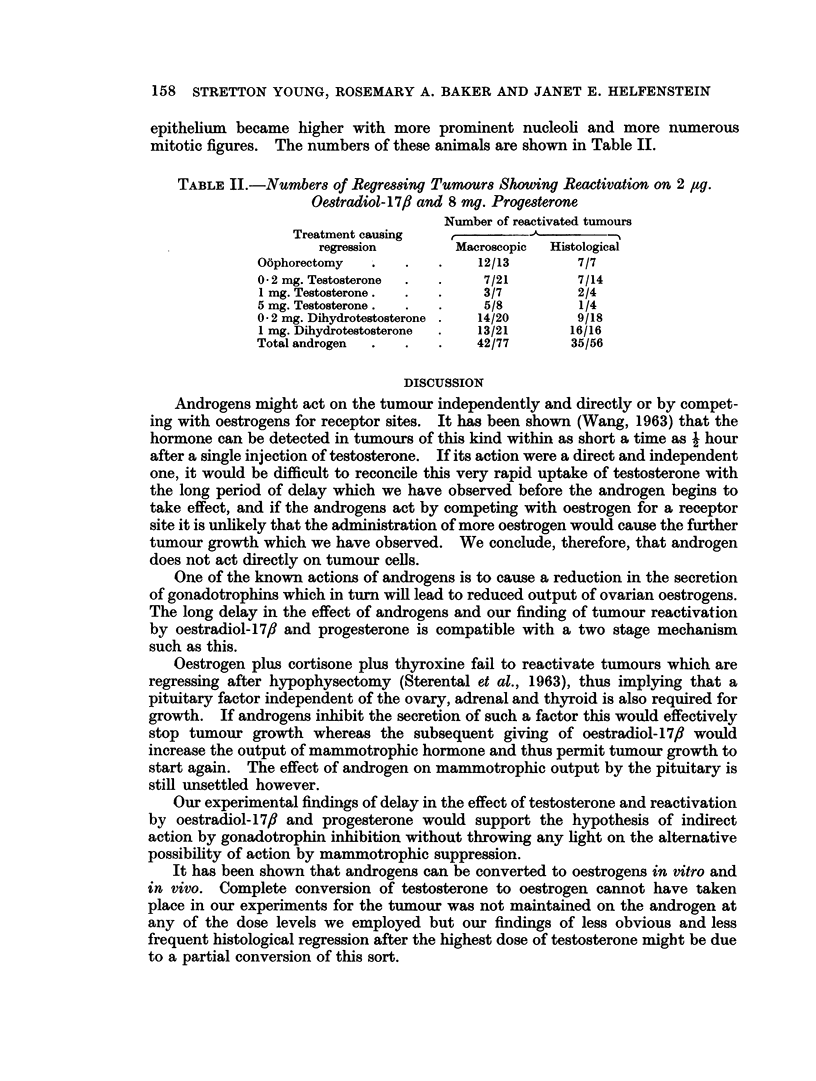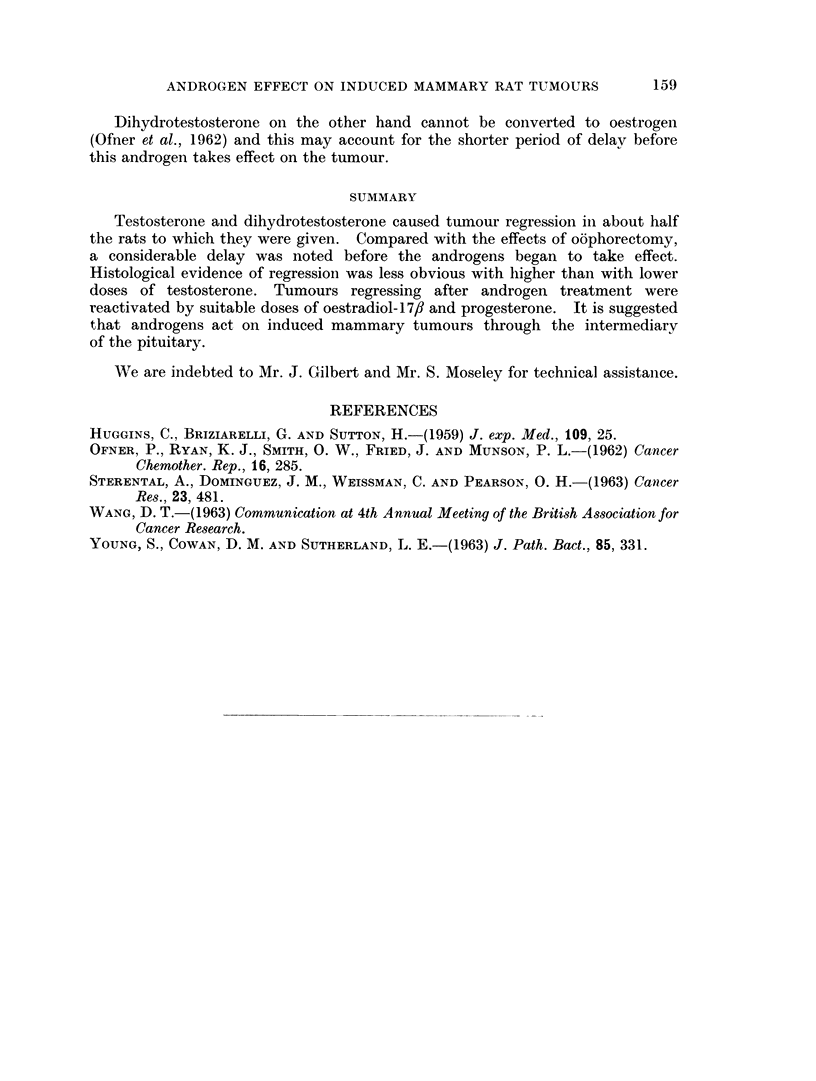# The Effects of Androgens on Induced Mammary Tumours in Rats

**DOI:** 10.1038/bjc.1965.18

**Published:** 1965-03

**Authors:** Stretton Young, Rosemary A. Baker, Janet E. Helfenstein


### THE EFFECTS OF ANDROGENS ON INDUCED MAMMARY TUMOURS IN RATS

STRETTON YOUNG, ROSEMARY A. BAKER AND JANET E. HELFENSTEIN

*From the Pathology Unit, Imperial Cancer Research Fund, London W.C.2*

Received for publication October 22, 1964

MAMMARY tumours induced in rats by the oral administration of 9,10-dimethyl-1,2-benzanthracene (DMBA) can be made to regress when the hormonal status is altered as, for example, by oöphorectomy, hypophysectomy or androgen administration (Huggins Briziarelli and Sutton, 1959). The exact means by which all of these forms of hormonal interference act on the tumour are not yet fully understood. In this paper we have compared the effects of oöphorectomy and varying doses of two androgens, testosterone and 17$\beta$ hydroxy-5$\alpha$ androstan-3-one (dihydrotestosterone) on tumours of this type.

### MATERIAL AND METHODS

Non-inbred female rats, from stock imported from Sprague-Dawley of Madison, Wisconsin, and bred commercially in Great Britain, were used throughout. They were maintained on diet G.R. 25 with water *ad libitum*. At 50 $\pm$ 1 days they were given intragastrically a single dose of DMBA dissolved in 2 ml. of corn oil. The majority were given 30 mg. of DMBA but in some cases a dose of 50 mg. was used.

Starting 4 weeks after the carcinogen was given the rats were examined twice weekly for the presence of tumours. When these were large enough they were measured with calipers in two directions, one in the long axis of the tumour and the other at right angles to it; the mean of the two diameters was used as our measure of tumour size.

When it was seen from the measurements that the tumours were growing steadily, oöphorectomy was carried out or androgen injections were given. Androgens and other hormones were given subcutaneously dissolved in corn oil for 6 days per week in daily doses as follows :

| | | |
|---|---|---|
| Testosterone | 0·2mg. | in 0·2 ml. oil |
| | 1 mg. | in 0·2 ml. oil |
| | 5 mg. | in 1 ml. oil |
| Dihydrotestosterone | 0·2mg. | in 0·2 ml. oil |
| | 1 mg. | in 0·2 ml. oil |
| Oestradiol-17$\beta$ | 2 $\mu$g. | together in 0·4 ml. oil |
| Progesterone | 8 mg. | together in 0·4 ml. oil |

Pieces of tumour removed at biopsy or autopsy were fixed in Bouin's fluid and 10 per cent neutral buffered formalin (4 per cent formaldehyde) and embedded in paraffin. Sections were cut at 4$\mu$ and stained with haematoxylin and eosin. Two hundred and thirty-two tumours from 189 rats were examined.

### RESULTS

The effects of giving androgens resembled the effects of oöphorectomy in that each was followed by tumour regression and the accompanying histological changes were similar to one another (Huggins *et al.*, 1959; Young, Cowan and Sutherland, 1963). There was one important difference, however, between the effects of the two forms of treatment. Whereas oöphorectomy caused a reduction in tumour size which could be detected within 2 days, the administration of testosterone permitted tumour growth to continue for up to 8 days. The mean growth curves of 42 tumours regressing after oöphorectomy and 28 tumours regressing after 1 mg. of testosterone per rat per day are compared in Fig. 1.

With increasing doses of testosterone the period of delay diminished slightly but it was clear that even with a very large dose of 5 mg. testosterone per rat per day, immediate regression such as was seen after oöphorectomy did not often occur. The period of delay was considerably less on dihydrotestosterone, particularly at the higher dose level. Delay was not related to tumour size at the start of treatment. The proportions of tumours undergoing regression were not significantly different with any of the treatments we employed, but it was our impression that the number of tumours showing histological regression and the degree of histological regression was considerably less on 5 mg. testosterone than at the two smaller dose levels. These results are summarized in Table I.

TABLE I.—*Numbers of Tumours Undergoing Regression*

| Treatment | Number of regressing tumours: Macroscopic | Number of regressing tumours: Histological | Number of tumours with delay before regression | Mean delay (days) |
|---|---|---|---|---|
| Oöphorectomy | 43/53 | 18/22 | 5/43 | $0 \cdot 5 \pm 0 \cdot 2$ |
| 0·2 mg. Testosterone | 21/33 | 15/28 | 17/21 | $9 \cdot 2 \pm 1 \cdot 5$ |
| 1 mg. Testosterone | 28/44 | 17/38 | 24/28 | $6 \cdot 4 \pm 0 \cdot 7$ |
| 5 mg. Testosterone | 18/29 | 5/22 | 16/18 | $5 \cdot 6 \pm 1 \cdot 0$ |
| 0·2 mg. Dihydrotestosterone | 24/35 | 15/31 | 13/24 | $4 \cdot 4 \pm 1 \cdot 0$ |
| 1 mg. Dihydrotestosterone | 28/38 | 20/33 | 15/28 | $2 \cdot 2 \pm 0 \cdot 6$ |
| Total androgen | 119/179 | 72/152 | 85/119 | |

Delay in the start of regression following androgen therapy can be explained in various ways. Possibly androgens do not act directly on the tumour but depress the output of pituitary hormones which may, in turn, reduce the secretion of ovarian steroids If this hypothesis is true it should be possible to reactivate tumours which are regressing on testosterone by the further administration of oestradiol-17$\beta$ and progesterone in doses which have caused further tumour growth following oöphorectomy Rats on testosterone whose tumours were regressing, were given oestradiol-17$\beta$ and progesterone, 2 $\mu$g and 8 mg. respectively per day. Forty-two of the 77 tumours in rats so treated increased in size and in about $\frac{3}{4}$ of them we found histological signs which we associate with reactivation. The

FIG. 1.—The mean growth curves of 42 tumours regressing after oöphorectomy and 28 tumours regressing after 1 mg. of testosterone per rat per day.

epithelium became higher with more prominent nucleoli and more numerous mitotic figures. The numbers of these animals are shown in Table II.

TABLE II.—*Numbers of Regressing Tumours Showing Reactivation on 2 μg. Oestradiol-17β and 8 mg. Progesterone*

| Treatment causing regression | Number of reactivated tumours | |
|---|---|---|
| | Macroscopic | Histological |
| Oöphorectomy | 12/13 | 7/7 |
| 0·2 mg. Testosterone | 7/21 | 7/14 |
| 1 mg. Testosterone | 3/7 | 2/4 |
| 5 mg. Testosterone | 5/8 | 1/4 |
| 0·2 mg. Dihydrotestosterone | 14/20 | 9/18 |
| 1 mg. Dihydrotestosterone | 13/21 | 16/16 |
| Total androgen | 42/77 | 35/56 |

### DISCUSSION

Androgens might act on the tumour independently and directly or by competing with oestrogens for receptor sites. It has been shown (Wang, 1963) that the hormone can be detected in tumours of this kind within as short a time as ½ hour after a single injection of testosterone. If its action were a direct and independent one, it would be difficult to reconcile this very rapid uptake of testosterone with the long period of delay which we have observed before the androgen begins to take effect, and if the androgens act by competing with oestrogen for a receptor site it is unlikely that the administration of more oestrogen would cause the further tumour growth which we have observed. We conclude, therefore, that androgen does not act directly on tumour cells.

One of the known actions of androgens is to cause a reduction in the secretion of gonadotrophins which in turn will lead to reduced output of ovarian oestrogens. The long delay in the effect of androgens and our finding of tumour reactivation by oestradiol-17β and progesterone is compatible with a two stage mechanism such as this.

Oestrogen plus cortisone plus thyroxine fail to reactivate tumours which are regressing after hypophysectomy (Sterental *et al.*, 1963), thus implying that a pituitary factor independent of the ovary, adrenal and thyroid is also required for growth. If androgens inhibit the secretion of such a factor this would effectively stop tumour growth whereas the subsequent giving of oestradiol-17β would increase the output of mammotrophic hormone and thus permit tumour growth to start again. The effect of androgen on mammotrophic output by the pituitary is still unsettled however.

Our experimental findings of delay in the effect of testosterone and reactivation by oestradiol-17β and progesterone would support the hypothesis of indirect action by gonadotrophin inhibition without throwing any light on the alternative possibility of action by mammotrophic suppression.

It has been shown that androgens can be converted to oestrogens *in vitro* and *in vivo*. Complete conversion of testosterone to oestrogen cannot have taken place in our experiments for the tumour was not maintained on the androgen at any of the dose levels we employed but our findings of less obvious and less frequent histological regression after the highest dose of testosterone might be due to a partial conversion of this sort.

Dihydrotestosterone on the other hand cannot be converted to oestrogen (Ofner *et al.*, 1962) and this may account for the shorter period of delay before this androgen takes effect on the tumour.

SUMMARY

Testosterone and dihydrotestosterone caused tumour regression in about half the rats to which they were given. Compared with the effects of oöphorectomy, a considerable delay was noted before the androgens began to take effect. Histological evidence of regression was less obvious with higher than with lower doses of testosterone. Tumours regressing after androgen treatment were reactivated by suitable doses of oestradiol-17$\beta$ and progesterone. It is suggested that androgens act on induced mammary tumours through the intermediary of the pituitary.

We are indebted to Mr. J. Gilbert and Mr. S. Moseley for technical assistance.

### REFERENCES

Huggins, C., Briziarelli, G. and Sutton, H.—(1959) *J. exp. Med.*, **109**, 25.

Ofner, P., Ryan, K. J., Smith, O. W., Fried, J. and Munson, P. L.—(1962) *Cancer Chemother. Rep.*, **16**, 285.

Sterental, A., Dominguez, J. M., Weissman, C. and Pearson, O. H.—(1963) *Cancer Res.*, **23**, 481.

Wang, D. T.—(1963) *Communication at 4th Annual Meeting of the British Association for Cancer Research.*

Young, S., Cowan, D. M. and Sutherland, L. E.—(1963) *J. Path. Bact.*, **85**, 331.